# Lie Group Methods in Blind Signal Processing

**DOI:** 10.3390/s20020440

**Published:** 2020-01-13

**Authors:** Dariusz Mika, Jerzy Jozwik

**Affiliations:** 1 Institute of Technical Sciences and Aviation, The State School of Higher Education in Chelm, 22-100 Chelm, Poland; 2 Faculty of Mechanical Engineering, Lublin University of Technology, 20-618 Lublin, Poland

**Keywords:** geometric optimization, Independent Component Analysis, independent subspace analysis, Lie groups, Lie algebra, toral subalgebra, sensors

## Abstract

This paper deals with the use of Lie group methods to solve optimization problems in blind signal processing (BSP), including Independent Component Analysis (ICA) and Independent Subspace Analysis (ISA). The paper presents the theoretical fundamentals of Lie groups and Lie algebra, the geometry of problems in BSP as well as the basic ideas of optimization techniques based on Lie groups. Optimization algorithms based on the properties of Lie groups are characterized by the fact that during optimization motion, they ensure permanent bonding with a search space. This property is extremely significant in terms of the stability and dynamics of optimization algorithms. The specific geometry of problems such as ICA and ISA along with the search space homogeneity enable the use of optimization techniques based on the properties of the Lie groups O(n) and SO(n). An interesting idea is that of optimization motion in one-parameter commutative subalgebras and toral subalgebras that ensure low computational complexity and high-speed algorithms.

## 1. Introduction

Blind signal processing (BSP) is currently one of the most attractive and fast-growing signal processing areas with solid theoretical foundations and many practical applications. BSP has become a vital research topic in many areas of application, particularly in biomedical engineering, medical imaging, speech and image recognition, communication systems, geophysics, economics, and data analysis. The term “blind processing” originates from the basic feature of these processing methods, i.e., the fact that there is no need to use any training data or a priori knowledge to obtain results. These methods include, among others, Independent Component Analysis (ICA), independent subspace analysis (ISA), sparse component analysis (SCA), nonnegative matrix factorization (NMF), singular value decomposition (SVD), principal component analysis (PCA) and minor component analysis (MCA) as well as the related eigenproblem and invariant subspace problem. Optimization problems of this kind often occur in the context of artificial neural networks, signal processing, pattern recognition, computer vision and numeric [1]. BSP is widely used in biomedical engineering, in technical diagnostics as well as in energy. The work [2] presents the use of SCA to analyze biomedical EEG and fMRI signals proving the effectiveness of this method in the detection of ocular artifacts. The use of SCA in technical diagnostics is presented in [3]. The three-dimensional geometric features-based SCA algorithm was used for compound faults diagnosis of roller bearing. A similar topic was discussed in [4] where NMF was used to extract error signals. The conducted experiments confirmed the effectiveness of these methods in extract the fault features and diagnosis for roller bearing. An interesting use of BSP techniques in energy issues is presented in [5]. Bayesian-optimized bidirectional Long Short-Term Memory (LSTM) method was used for energy disaggregation aiming to identify the individual contribution of appliances in the aggregate electricity load. The use of machine learning techniques as *k*-means clustering and Support Vector Machine for low-complexity energy disaggregation is presented in [6].

This paper primarily focuses on ICA and ISA problems, which does not, however, limit the applicability of the described methods to other types of problems. The scope of this paper is mainly limited to presenting the geometry of ICA and ISA problems and the application of Lie groups and Lie algebra without providing specific algorithms.

Standard Independent Component Analysis (ICA) consists of the linear transformation of multidimensional data such that the transformed signal components are as much statistically independent as possible. The effectiveness of ICA depends on the correct choice of a cost function and an optimization strategy. Most numerical optimization techniques assume that the model’s parameter space is a usual Euclidean space. In many cases, however, the parameter space has a non-linear structure with its unique non-Euclidean geometry. From a mathematical point of view, the space of search equipped with an inner product takes on the properties of Riemannian manifolds, often with desired algebraic properties [7].

The authors of works in this field take advantage of the specific internal geometry and algebraic properties of models such as the orthogonal group O(n) or the special orthogonal group SO(n). Apart from the general group properties, these groups also have the structure of a smooth differential manifold, and thus acquire the Lie group properties and the corresponding Lie algebra. The application of this convenient structure to ICA algorithms is described in [8,9].

From the point of view of standard optimization techniques, in issues of this type one deals with the so-called constrained optimization. The problem of constrained optimization occurs in many issues related to signal processing. In the case of ICA, optimization of this kind consists of looking for extrema of the cost function on the set of matrices satisfying the condition of orthonormal columns ($W^{T}W=I$). However, with standard constrained algorithms one operates in Euclidean space, so in each iterative step the matrix orthogonality is lost. To restore the orthogonality condition, it is necessary to perform orthogonalization in each iterative step (e.g., by the well-known Gram-Schmidt orthogonalization process), which, however, reduces the convergence rate of the algorithms. Other algorithms use the Lagrangian method of optimization by the addition to the cost function of the so-called penalty function to prevent an orthogonality deviation. However, such algorithms are characterized by a low convergence rate and poor quality of the achieved optimum.

If there is a limitation in the form of matrix orthogonality, one can use an alternative method that ensures “locked” with the hyper-surface of orthogonal matrices during optimization motion. This method uses the group structure of a set of orthogonal square matrices which, apart from the properties of a smooth differential manifold, provides the set with the properties of a special structure known as a Lie group.

## 2. Model Definition (ICA, ISA)

Standard independent components analysis (ICA) consists of estimating a sequence of *p* statistically independent components (ICs) $s_{1},\ldots ,s_{p}$ and the mixing matrix ***A*** of dimension n×p with only a sequence of *n* observed signals $x_{1},\ldots ,x_{n}$. Giving the source signals and observed signals in the form of the source vector $s={\left(s_{1},\ldots ,s_{p}\right)}^{T}$ and the vector of observed (mixed) signals $x={\left(x_{1},\ldots ,x_{n}\right)}^{T}$, where *T* stands for transposition, the standard linear ICA model takes the form (1):

(1) x=As

**Figure 1:**
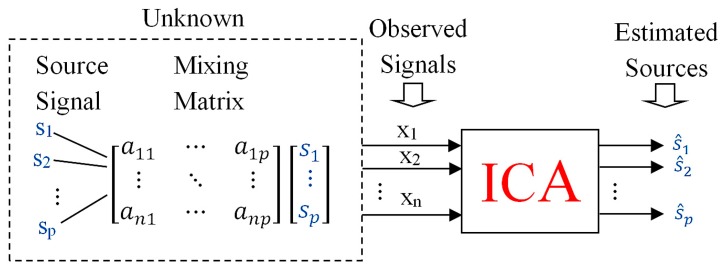
Schematic block scheme of Independent Component Analysis.

This assumes that there is no additional noise signal in the observed signal (Figure 1). The ICA model thereby formulated is characterized by a scale and permutation ambiguity, i.e., it is possible to scale (multiply by a given constant) of any source signals $s_{i}$ and at the same time to divide the *i*-th column $a_{i}$ of the mixing matrix A by this constant, while the observed signal x remains unchanged. The same phenomenon will occur at random transposition of any rows of the source vector s (permutation of the source vector s) and the same transposition of the columns of the mixing matrix A. It is customary to assume that the source signals have the unit variance ($C_{s}=E\left(ss^{T}\right)=I$). In non-negative ICA, it is additionally assumed that the source signals $s_{i}$ satisfy the condition $s_{i}\geq 0$ [10,11].

A solution for the ICA problem when n=p consists of finding the demixing (filtration) matrix $Q^{T}=A^{-1},\;Q\in Gl\left(n\right)$ where the filtration matrix Q belongs to a general linear group Gl(n) of non- singular matrices det(Q)≠0. Source signals are obtained via (2):

(2) $$\hat{s}=Q^{T}x=Q^{T}As$$

where $\hat{s}$ is the estimator of a source vector s (it meets the statistical assumptions for a source signal).

To reduce the computation load in ICA, the pre-processing usually involves performing the whitening of the observed signal to obtain the signal z=Bx=BAs, where B is the whitening matrix, with unit variance and the decorrelation $C_{z}=E\left(zz^{T}\right)=I$. Assuming that $C_{s}=I$, we get (3):

(3) $$I=C_{z}=E\left(zz^{T}\right)=BAE\left(ss^{T}\right){\left(BA\right)}^{T}=BA{\left(BA\right)}^{T}$$

From this it follows that ${\left(BA\right)}^{T}={\left(BA\right)}^{-1}$. Hence, the transformation from s to z takes place via an orthogonal matrix BA. Therefore, if $\hat{s}=W^{T}z=W^{T}BAs=Us$, then the matrix $U=W^{T}BA$ must be an orthogonal matrix (permutation matrix), and thus a new filtering matrix W (after whitening) must also satisfy the orthogonality condition. The whitening of the observed signal therefore simplifies the ICA problem from optimization on the general linear group Gl(n) (matrices Q only satisfying the invertibility condition det(Q)≠0) to optimization on the special orthogonal group SO(n) (matrices W satisfying the orthogonality condition $W^{T}W=I$). Both groups are Lie groups at the same time.

Standard ICA is based on the assumption that n=p, i.e., the number of source signals $s_{i}$ is known and equal to the number of observed signals $x_{i}$. ICA also yields interesting results in a more general case when the number of estimated source signals *p* is unknown. In this case, it can be n≠p. When n<p, i.e., the number of observed signals is smaller than the number of source signals, the problem is known as over complete bases ICA, whereas when n>p it is called under complete bases ICA. This kind of problem can be formally considered to be unconstrained optimization on the Stiefel manifold [12,13]. It is also possible to solve ICA problems for the case p=1. This type of problem is often called Single Channel Source Separation [14,15].

Hyvarinen and Hoyer introduced independent subspace analysis (ISA) [16] by omitting the statistical independence condition between extracted source components. The source vector s is composed in $d_{k}$-tuple (k=1,…,r), where for a given tuple a statistical dependence between its source signals $s_{i}$ is allowed, while signals belonging to different tuples are statistically independent. When using the whitening process, the ISA problem boils down to finding orthogonal matrices $W^{T}W=I$ as in standard ICA. However, due to the statistical relationship between the source signals in the tuple, ISA problem optimization cannot be performed on an ordinary Stiefel manifold. It is necessary to introduce a different, more universal manifold allowing for additional symmetries. This manifold is known as a flag manifold.

Traditionally, the ICA model assumes the statistical independence of extracted source signals. It turns out, however, that there are reasons to replace the orthonormality condition with the condition of source signal normality [17]. A precise definition of the ICA problem consists of finding a linear non-orthogonal transformation (of the coordinate system) of multidimensional data such that the transformed data have minimal mutual information. Hyvarinen [18] demonstrated (in an ICA problem) the differences between the use of cost functions based on mutual information and those based on the so-called non-Gaussianity. Achieving maximum de-correlation by maximizing the sum of non-Gaussianity of independent components (ICs) is not necessarily related to the minimization of mutual information (MI). In addition, the orthonormality condition leads to a smaller subset of matrices, which simplifies the optimization process yet may reduce its quality. Orthonormality imposes a greater limitation on the degrees of freedom than normality. In standard ICA, the orthonormality condition of n×n filtering matrices reduces the number of the degrees of freedom to (n−1)/2, while the normality condition increases the number of free parameters to n(n−1), which considerably improves the quality of obtained results. A problem of this type can be formally considered to be the unconstrained optimization of an oblique manifold [19,20].

## 3. Geometry of ICA, ISA and Other BSP Models

The manifolds frequently arise from BSP tasks for a general do not have the group properties. Nevertheless, they are homogenous spaces of the Lie groups. A homogenous space M is a manifold on which the Lie group G acts transitively [21]. This property is fundamental for the considered manifolds because it enables analyzing them as quotient spaces. As mentioned in Section 2, the optimization problem in standard ICA (ISA) boils down to optimizing on the general linear group Gl(n) (matrices Q only satisfy the invertibility condition det (Q)≠0). The whitening of the observed signal simplifies the ICA problem to optimization on the special orthogonal group SO(n) (matrices W satisfy the orthogonality condition $W^{T}W=I_{n}$). In the case of an under complete problem p<n, i.e., when the number of extracted ICs is smaller than the number of observed signals, the set of filtering matrices can be treated as an orthogonal Stiefel manifold St(n,p) defined as the set of orthonormal matrices of dimension n×p with the form (4):

(4) $$St\left(n,p\right)=\{W=\left(w_{1},\ldots ,w_{p}\right)|W\in R^{n\times p},\;W^{T}W=I_{p},rank\left(W\right)=p\}$$

which can be regarded as the quotient space arising from the orthogonal group.

Lie group G=O(n) acts transitively on the Stiefel manifold via (5):

(5) O(n)×St(n,p)∋(Q,W)→QW∈St(n,p)

where Q∈O(n), W∈St(n,p). It is possible to demonstrate that for two given points $W_{1},\;W_{2}\in St\left(n,p\right)$ there exists Q∈O(n) such that $W_{2}=QW_{1}$. This means that starting from any point $W_{0}\in St\left(n,p\right)$ it is possible to reach any point W∈St(n,p) by the G action. Resorting to group theory terminology, one can say that the entire manifold St(n,p) is equivalent to the single orbit $G(W_{0})$ of a given point $W_{0}$ where

(6) $$G(W_{0})=\left\{W=QW_{0}|Q\in O\left(n\right),\;W_{0}\in St\left(n,p\right)\right\}$$

The point W on the manifold St(n,p) can be expressed via a certain point Q on O(n)**.** The mapping π: Q→W is surjective, i.e., many to one (projective mapping). Redundancy of this mapping is described by so-called the isotropy subgroup H of the point $W_{0}$. It is a set of matrices that do not change $W_{0}$

(7) $$H=\left\{N\in O\left(n\right)|\;NW_{0}=W_{0}\right\}$$

The isotropy subgroup H∈O(n) of the group O(n) of the point $W_{0}\in St\left(n,p\right)$ has the form (8):

(8) $$H=\left(W_{0},W_{0}{}_{⊥}\right)\left(\begin{array}{cc} I_{p} & 0\\ 0 & O\left(n-p\right) \end{array}\right){\left(W_{0},W_{0}{}_{⊥}\right)}^{T}$$

where $W_{0}{}_{⊥}\in St\left(n,n-p\right)$ is any n×(n−p) matrix that satisfies the condition $\left(W_{0},W_{0}{}_{⊥}\right)\in O\left(n\right)$. It is easy to check that the isotropy condition of point $W_{0}$ is satisified (9):

(9) $$H\cdot W_{0}=\left(W_{0},W_{0}{}_{⊥}\right)\left(\begin{array}{cc} I_{p} & 0\\ 0 & O\left(n-p\right) \end{array}\right){\left(W_{0},W_{0}{}_{⊥}\right)}^{T}W_{0}=W_{0}$$

Choosing $W_{0}=\left(\begin{array}{c} I_{p}\\ 0_{n-p,p} \end{array}\right)$ the isotropy subgroup $W_{0}$ is a set $H=\left(\begin{array}{cc} I_{p} & 0\\ 0 & O\left(n-p\right) \end{array}\right)$.

In this shot two n × n orthogonal matrices represent the same point of the Stiefel manifold if their first *p* columns are identical or equivalently, if they are related by right multiplication of a matrix of the form $\left(\begin{array}{cc} I_{p} & 0\\ 0 & O\left(n-p\right) \end{array}\right)$ where O(n−p) is an orthogonal matrix group of dimension (n−p)×(n−p) [1]. From a mathematical point of view, we say that such representations are in an equivalence relation. All matrices in an equivalence relation form what is called the equivalence class [W]. Thus, the point on the Stiefel manifold is the equivalence class [W] of n×n orthogonal matriceswith identical first *p* columns, while the Stiefel manifold is a quotient space of the form (10):

(10) St(n,p)≅O(n)/O(n−p)

and specifically as St(n,p)≅O(n)/H where $H=\left(\begin{array}{cc} I_{p} & 0\\ 0 & O\left(n-p\right) \end{array}\right)$. However, H is isomorphic to O(n−p), i.e., H≅O(n−p), therefore St(n,p)≅O(n)/O(n−p).

There are many applications for the problem formulated as the finding of (zero) extreme of a given field defined in a non-Euclidean subspace of dimension p embedded in the Euclidean space $R^{n}$. This non-Euclidean subspace is known as the Grassmann manifold Gr(n,p;R) [22,23]. Grassmann manifolds can be described as an equivalence class of n×p orthogonal matrices spanning the same *p*-dimensional subspace [W]={WO(p)|W∈St(n,p)}. Therefore, from a theoretical point of view, the Grassmann manifold can be expressed as the quotient space Gr(n,p)≅St(n,p)/O(p) and given that St(n,p)≅O(n)/O(n−p), the Grassmann manifold Gr(n,p) can also be seen as the quotient space O(n)/O(p)×O(n−p). In this case, the equivalence class $\left[W\right]=\{W\left(\begin{array}{cc} O_{p} & 0\\ 0 & O\left(n-p\right) \end{array}\right)|W\in O\left(n\right)\}$ is a set of square *n*-dimensional orthogonal matrices whose first *p* columns span the same *p*-dimensional subspace. Manifolds of this type are used, among others, in invariant subspace analysis, application-driven dimension reduction and subspace tracking [24,25].

When there is a need for a simultaneous (parallel) subspace extraction, as is the case in independent subspace analysis (ISA), one resorts to the concept of generalized flag manifold, which is a manifold consisting of orthogonal subspaces that constitutes a generalization of both Stiefel and Grassmann manifolds [26,27,28]. The generalized flag manifold $Fl\left(n,d_{1},\ldots ,d_{r};R\right)$ is defined as (11):

(11) $$Fl\left(n,d_{1},\ldots ,d_{r};R\right)=\{W|W\in R^{n\times p},\;W^{T}W=I_{p},\}$$

where the orthogonal matrix W takes the form (12):

(12) $$W=\left[W_{1},\ldots ,W_{r}\right],W_{i}=\left[w_{1}^{i},\ldots ,w_{d_{i}}^{i}\right],$$

where $w_{k}^{i}\in R^{n},\;k=1,\ldots ,d_{r}$ for a specified i=1,…,r is a set of orthogonal bases that span subspaces $V_{i}$. The subspaces $V_{i}$ are orthogonal relative to each other and satisfy the condition (13):

(13) $$V=V_{1}⊕V_{2}⊕\ldots ⊕V_{r}\subset R^{n\times p}$$

Points on the flag manifold are a set of vector spaces V which can be decomposed as (13). If all $d_{i}\left(1\leq i\leq r\right)=1$, the manifold $Fl\left(n,d_{1},\ldots ,d_{r};R\right)$ is reduced to the Stiefel manifold St(n,p). If r=1, it is reduced to the Grassmann manifold Gr(n,p). It is abbreviated as Fl(n,d) where $d=(d_{1},\ldots ,d_{r})$. The orthogonal group O(n) also acts transitively on the manifold Fl(n,d) via simple matrix multiplication (14):

(14) O(n)×Fl(n,d)∋(Q,W)→QW∈Fl(n,d)

The isotropy subgroup H∈O(n) of the group O(n) of the point W∈Fl(n,d) has the form (15):

(15) $$H=\left(W,W_{⊥}\right)diag\left(R_{1},\ldots ,R_{r},R_{r+1}\right){\left(W,W_{⊥}\right)}^{T}$$

where $diag\left(R_{1},\ldots ,R_{r},R_{r+1}\right)$ is a block-diagonal matrix of the form $\left(\begin{array}{cccc} R_{1} & & & 0\\ & \ddots & & \vdots \\ \vdots & & R_{r} & \\ 0 & & & R_{r+1} \end{array}\right)$, $R_{k}\in O\left(d_{k}\right),\;\left(1\leq k\leq r\right),\;R_{r+1}\in O\left(n-p\right)$, $W_{⊥}\in St\left(n,n-p\right)$ is any n×(n−p) matrix that satisfies the condition $\left[W,W_{⊥}\right]\in O\left(n\right)$. It is easy to check that the isotropy condition of point W is satisified (16):

(16) $$\begin{array}{c} H\cdot W=\left[W\right]\left(W,W_{⊥}\right)diag\left(R_{1},\ldots ,R_{r},R_{r+1}\right){\left(W,W_{⊥}\right)}^{T}\cdot W=\left(WR,\;W_{⊥}R_{r+1}\right)\left(W^{T}W_{⊥}^{T}\right)W\\ =\;\left(WRW^{T}+W_{⊥}R_{r+1}W_{⊥}^{T}\right)W=WR=\left[W\right] \end{array}$$

where $R=diag\left(R_{1},\ldots ,R_{r}\right)$, $WR=\left[W\right]=diag\left(W_{i}R_{i}\right),\;i=1,\ldots ,r$ is an equivalence class of the point on Fl(n,d). This means that any two matrices $W_{1}$ and $W_{2}$ satisfying the condition $W_{2}=W_{1}R=\left(W_{1},\ldots ,W_{r}\right)diag\left(R_{1},\ldots ,R_{r}\right)=diag\left(W_{1}R_{1},W_{2}R_{2}\;,\ldots ,W_{r}R_{r}\right)$ are identified with the very same point on the manifold Fl(n,d). Given the above,

(17) $$Fl\left(n,d\right)≅O\left(n\right)/O\left(d_{1}\right)\times \ldots \times O\left(d_{r}\right)\times O\left(n-p\right)$$

As it was already mentioned, the manifold Fl(n,d) is locally isomorphic to St(n,p) as a homogenous space when all $d_{i}\left(1\leq i\leq r\right)=1$ and to the manifold Gr(n,p) when r=1.

In terms of optimization, the homogeneity of the considered differential manifolds enables the search (optimization motion) in the group O(n) or SO(n), and the use of optimization techniques that are well known and adapted to these types of groups. Section 4 presents the basic ideas of optimization methods used in SO(n) and the concept of toral subalgebra that is characteristic of problems of this type.

## 4. Lie Group Optimization Methods. One-Parameter Subalgebra and Toral Subalgebra

The idea of a standard optimization procedure based on the Lie groups consists of performing the optimization motion in the Lie algebra space and then mapping exp to find a solution in the Lie group (manifold). The optimization motion in the group SO(n) starting from the point (matrix) $W_{0}$ therefore consists of, first, the transition to the Lie algebra Ω=logW∈so(n) via mapping inversely to the exponentiation $\log:=\exp^{-1}$ of motionin the Lie algebra (performing an operation of addition (of matrices) in the abelian group) in order to obtain a new antisymmetric matrix $\Omega^{\prime }\in so\left(n\right)$ and, finally, returning to the Lie group via exponential mapping $W^{\prime }=\exp\;\Omega^{\prime }\in SO\left(n\right)$. A simple update method using line search procedure relies on finding the search direction in Lie algebra so(n) calculating the gradient of cost function J in Lie algebra space. This gradient must be skew-symmetric (see Appendix A) so (18) [9]:

(18) $$\nabla_{A}J=(\nabla_{w}J)\;W^{T}-W(\nabla_{w}J){\;}^{T}$$

Applying the steepest descent procedure with small constant update factor μ we start from $A=0_{n}\in so\left(n\right)$, move to $B=-\mu \nabla_{A}J$, map to R=exp(B)∈SO(n) and finally perform rotating (multiplicative) update $W_{k+1}=\exp\left(-\mu \nabla_{A}J\right)W_{k}$. This kind of optimization method is called a geodesic flow method [9].

At this point it is necessary to comment on motion in the Lie algebra. In our context, the addition of vectors in the Lie algebra so(n) can only be useful if it is matched by multiplication in the Lie group SO(n). Then one can write (19):

(19) exp(A)exp(B)=exp(B)exp(A)=exp(A+B)

As was already mentioned, this equation holds true only when the matrices A and B commutate [A,B]=0. This condition is satisfied for all matrices with so(2). When n≥3, this condition is not satisfied for all matrices in the algebra. When the matrices not commutate (non-abelian Lie algebra), Equation (19) is not satisfied and optimization motion in the Lie algebra (sum A+B) in a direction of e.g., the cost function gradient will not be reflected in the Lie group exp(A+B)≠exp(A)exp(B). However, taking $\exp\left(A\right)=I_{n}$, which is tantamount to selecting an initial matrix $A_{0}=0_{n}$, this condition will always be satisfied. In this case, $[A_{0},B]=0$, and Equation (19) is satisfied too. This is tantamount to motion in the one-parameter Lie algebra. By selecting A=tΩ for a random antisymmetric matrix Ω∈so(n) and a scalar ∈R, all matrices of this form commutate with each other ($A=t_{1}\Omega ,\;B=t_{2}\Omega :\left[A,B\right]=t_{1}\Omega t_{2}\Omega -t_{2}\Omega t_{1}\Omega =0$). A set of such matrices $so_{\Omega }\left(n\right)=\{t\Omega |\Omega \in so\left(n\right),\;t\in R\}\;$ is in itself a Lie algebra known as a one-parameter subalgebra of the Lie algebra so(n). The subalgebra $so_{\Omega }\left(n\right)$ is an abelian (commutative) algebra related to the one-parameter subgroup R(t)=exp(tΩ). Optimization motion in the subalgebra $so_{\Omega }\left(n\right)$ is therefore an equivalent (generalization) to the idea of linear motion in Euclidean space. In this case, the optimization procedure consists of searching for a minimum of the cost function along the subalgebra $so_{\Omega_{1}}\left(n\right)$ (for a chosen search direction $\Omega_{1}$), which corresponds to the search along the subgroup R(t).

Having found the cost function minimum $\left(R\left(t\right)W_{0}\right)$, where $W_{0}$ is a starting point, a new direction of linear searches $\Omega_{2}$ is selected, and the procedure is repeated until the desired convergence is achieved. Plumblay [8] proposed a modification of the standard procedure described above. This modification consists of moving the point of “origin” of the Lie algebra from a neutral element of the group to point W. Due to the group properties SO(n), it can be written that $W^{\prime }=RW$ for some matrix R∈SO(n). Moving from the matrix $W=I_{n}W$ to $W^{\prime }=RW$ is therefore equivalent to moving from the identity matrix $I_{n}$ to the matrix R. This procedure consists of moving from the matrix $0_{n}=\log I_{n}\in so\left(n\right)$ to Ω=logR∈so(n) in the Lie algebra and then returning to the group SO(n) via the exponential mapping R=expΩ and, finally, determining $W^{\prime }=RW=\left(\exp\Omega \right)W\in SO\left(n\right)$. This is equivalent to the concept of optimization motion in the one-parameter abelian subalgebra described above.

The above optimization procedures are computationally expensive due to the necessity of performing (computationally expensive) matrix exponentiation in every iterative step. The representation of antisymmetric matrices in the Jordan canonical form enables the decomposition of optimization movement in the group SO(n) to commutative rotations in orthogonal planes. Every antisymmetric matrix Ω can be presented in a block-diagonal form (for 2 m≤n) (20):

(20) $$\Omega =Qdiag\left(\Phi_{1},\ldots ,\Phi_{m},0,\ldots ,0\right)Q^{t}$$

where $diag\left(\Phi_{1},\ldots ,\Phi_{m},0,\ldots ,0\right)$ is a block-diagonal matrix of the form $\left(\begin{array}{cccccc} \Phi_{1} & & \ldots & & & 0\\ & \ddots & & & & \\ & & \Phi_{m} & & & \vdots \\ \vdots & & & 0 & & \\ & & & & \ddots & \\ 0 & & & & & 0 \end{array}\right)$, Q∈SO(n), $\Phi_{i}=\left(\begin{array}{cc} 0 & \varphi_{i}\\ -\varphi_{i} & 0 \end{array}\right)$ denotes the 2×2 dimensional antisymmetric matrices [29]. This form is known as the Jordan canonical form. Since the relationship $\exp\left(Q^{T}\Omega Q\right)=Q^{T}\exp\left(\Omega \right)Q$ holds true, the matrix Ω can be decomposed into a sum of the form $\Omega =\Omega_{1}+\ldots +\Omega_{m}$ where $\Omega_{i}$ is the matrix only containing the *i*-th Jordan matrix $\Phi_{i}$ and zeros beyond it (21):

(21) $$\Omega =Qdiag\left(\Phi_{1},0,\ldots ,0\right)Q^{t}+\ldots +Qdiag\left(0,\ldots ,\Phi_{m},0,\ldots ,0\right)Q^{t}$$

The exponentiation of thereby presented matrix Ω yields an orthogonal matrix W of the form (22):

(22) $$W=\exp\Omega =Qdiag\left(R_{1},\ldots ,R_{m},1,\ldots ,1\right)Q^{t}$$

where $R_{i}=\left(\begin{array}{cc} cos\varphi_{i} & sin\varphi_{i}\\ -sin\varphi_{i} & cos\varphi_{i} \end{array}\right)$ are the 2×2 dimensional rotation matrices. The matrix W can be decomposed into a product of the matrix $W=W_{1}\ldots W_{m}$ where $W_{i}$ has the form (23):

(23) $$W_{i}={\exp\Omega }_{i}=Qdiag\left(1,\ldots ,1,R_{i},1,\ldots ,1\right)Q^{t}$$

One can notice that the exponentiation of the matrix Ω in the Jordan form is reduced to a simple and inexpensive calculation of the functions $sin\varphi_{i}$ and $cos\varphi_{i}$, which significantly increases the speed of optimization algorithms. The Jordan canonical form of antisymmetric matrix can be obtained via symmetric eigenvalue decomposition [29]. It can be observed that the antisymmetric matrix Ω commutates with the symmetric matrix $\Omega^{2}=-\Omega^{T}\Omega$, which means that Ω and $\Omega^{T}\Omega$ have the same eigenvectors and eigenvalues. The eigenvalues $\Omega^{T}\Omega$ occur in pairs corresponding to individual Jordan matrices $\Phi_{i}$.

This form can be visualized as compounding rotations (represented by $W_{i}$) in mutually orthogonal planes. In addition, the rotation matrices $W_{i}$ commutate $[W_{i},W_{j}]=0$. The commutation property of the rotation matrix $W_{i}$ provides the possibility of using the optimization procedure on SO(n), moving in the Lie algebra so(n).

The case of SO(4) is interesting from a geometrical point of view. The Jordan canonical form of the antisymmetric matrix Ω contains two blocks (matrices) $\Phi_{i}$:

(24) $$\Omega =Q\left(\begin{array}{cc} \begin{array}{cc} 0 & \varphi_{1}\\ -\varphi_{1} & 0 \end{array} & \begin{array}{cc} 0 & \;0\\ 0 & \;0 \end{array}\\ \begin{array}{cc} 0 & \;0\\ 0 & \;0 \end{array} & \begin{array}{cc} 0 & \varphi_{2}\\ -\varphi_{2} & 0 \end{array} \end{array}\right)Q^{t}$$

Here, the orthogonal matrix W takes the form (25):

(25) $$W=\exp\Omega =Q\left(\begin{array}{cc} \begin{array}{cc} cos\varphi_{1} & sin\varphi_{1}\\ -sin\varphi_{1} & cos\varphi_{1} \end{array} & \begin{array}{cc} 0 & \;0\\ 0 & \;0 \end{array}\\ \begin{array}{cc} 0 & \;0\\ 0 & \;0 \end{array} & \begin{array}{cc} cos\varphi_{2} & sin\varphi_{2}\\ -sin\varphi_{2} & cos\varphi_{2} \end{array} \end{array}\right)Q^{t}$$

**Figure 2:**
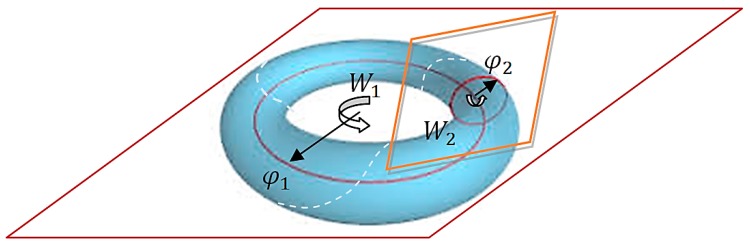
Visual representation of the toral subalgebra t(p) for p=2. The angles $\varphi_{1}$, $\varphi_{2}$ and the matrices $W_{1}$ and $W_{2}$ are as in Equation (23). The broken line marks the search curve for the case $\frac{k_{1}}{k_{2}}=3$.

A visual representation of this case shows rotations in two mutually orthogonal planes, which corresponds to toral geometry (Figure 2).

From the point of view of optimization procedures, the rotation angles $\varphi_{1}$ and $\varphi_{2}$ should not be free parameters (independent of each other). For the procedure to make sense, the curve over which the search is carried out after a complete rotation (or its multiple) relative to one of the planes of rotation should return to the starting point on the toral surface (Figure 2). This is possible when for some t, the relationships $t\varphi_{1}=2k_{1}\pi$ and $t\varphi_{2}=2k_{2}\pi$ are satisfied for the integers $k_{1}$ and $k_{2}$. Therefore, the angle of rotation should be described by the relationship $\frac{\varphi_{1}}{\varphi_{2}}=k_{1}/k_{2}$ or $\varphi_{1}=a\varphi_{2}$, where $a=k_{1}/k_{2}$ is a rational number. This concept is naturally transferred to a general case of SO(n) for n>4. The Jordan canonical form represents optimization motion in one-parameter Lie subalgebra $R\left(t\right)=\exp\left(t\Omega \right)\in so_{\Omega }\left(n\right)$ as a rotation in p mutually orthogonal planes, and these rotations are commutative. The geometry of 2-dimensional torus for SO(4) can also be generalized to the geometry of the p-dimensional torus in SO(n) where n=2p for even n or n=2p+1 for odd n. This perception of motion in SO(n) leads to the concept of toral subalgebra t(p)⊂so(n). If we consider a general case of motion on the surface of a p-dimensional torus where the angles of rotation $\varphi_{i}$ are not interrelated by the above relationship, and individual independent planes of rotation are represented by a set p of commuting matrices $\Omega_{i}$ ($\Omega =\underset{i}{\sum }\Omega_{i}$). The motion (or rather rotation) on each of the independent planes of rotation can be expressed in the form of a parameterized curve $B_{i}=t_{i}\Omega_{i}$, or actually via its simple exponentiation $W_{i}=\exp(t_{i}\Omega_{i})$. The set of independent parameters $t_{i}$ that can be identified with the angles of rotation $\varphi_{i}$ forms a coordinate system on the toral subalgebra t(p). Compared to the original n–dimensional search space SO(n), the toral subalgebra t(p) is, however, an abelian algebra, which means that motion in this search space is commutative. This ensures the possibility of motion in all directions specified by the coordinates $t_{i}$ and their sum in the form $B=t_{1}\Omega_{1}+\ldots +t_{p}\Omega_{p}$ will be reflected in the composition of rotations $W=\exp B=\exp\left(t_{1}\Omega_{1}\right)\cdot \ldots \cdot \exp\left(t_{p}\Omega_{p}\right)$. The optimization procedure based on such a concept consists of decomposing to canonical form a specific antisymmetric matrix B∈so(n) (this can be, for example, a cost function gradient as in the method of steepest descent), and thereby formulating a toral subalgebra. Since the orthogonal matrix Q in the Jordan decomposition (24) is constant, the transition to a new point W in the search space is done by determining p values of the sin and cos functions corresponding to p planes of rotation. After finding in the subalgebra the point that minimizes the cost function, a new antisymmetric matrix $B^{\prime }\in so\left(n\right)$ is calculated and again presented in the Jordan canonical form, which establishes a new toral subalgebra. The procedure is repeated until the set minimum cost function is reached. A different problem concerns the determination of the direction of search B∈so(n) and the manner of search along the subalgebra. The selection of directions and the manner of searches depend on the adopted optimization procedure. It can be the steepest descent (SD) method and, in general, geodesic flow, Newton’s method or conjugate gradients. This problem has been extensively studied in [12,22,30].

## 5. Experimental Results

To illustrate the presented optimization methods on Lie groups, we will first present a rather simply simulation experiment. The purpose of this example is to show how different algorithms work on optimization problems with unitarity constraint. To this end, let us consider the Lie group of complex numbers with the unit module U(1), which is isomorphic with the SO(2) group. The unitarity constraint of elements of this group is a unit circle on the complex plane. The cost function we will minimize will be $J\left(z\right)={\left|z+0.3\right|}^{2}$ with constraint $zz^{*}=1$. For optimization, we will use five types of steepest descent (SD) algorithms:

(1) algorithm SD unconstrained on the Euclidean space,
(2) algorithm SD on the Euclidean space with constraint restoration,
(3) algorithm SD on the Euclidean space with penalty function,
(4) non-geodesic algorithm SD on Riemannian space,
(5) geodesic algorithm SD on Riemannian space.

**Figure 3:**
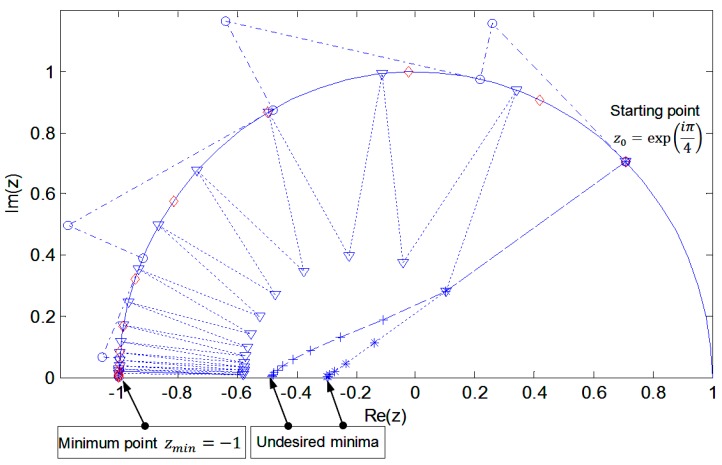
Comparison of SD algorithms for minimizing the cost function on group U(1). Methods in the Euclidean versus Riemannian space (Lie group methods). * algorithm SD unconstrained on the Euclidean space (1). 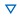 algorithm SD on the Euclidean space with constraint restoration (2). + algorithm SD on the Euclidean space with penalty function (3). o non-geodesic algorithm SD on Riemannian space (4). 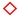 geodesic algorithm SD on Riemannian space (5).

In Algorithm (1) update rule has the form $z_{k+1}=z_{k}-\mu \left(z_{k}+0.3\right)$ where μ is the step size. The quantity $\frac{\partial J}{\partial z^{*}}=\left(z_{k}+0.3\right)$ is the gradient (The gradient of the function defined in the complex space has the form [31]:∂J∂z*=12(∂J∂Re(z)+∂J∂Im(z)) where Re(z) and Im(z) is respectively the real and imaginary part of a complex number z.) of the cost function J on the Euclidean space. Algorithm (2) uses the same update rule, but after each iteration the unitarity condition is restored in the form $z_{k+1}=\frac{z_{k+1}}{\left|z_{k+1}\right|}$. In Algorithm (3) we used the Lagrange multiplier method. The penalty function of the form ${\left({\left|z_{k}\right|}^{2}-1\right)}^{2}$ weighted by a Lagrange parameter λ has been added to the initial cost function in order to penalize deviations from unitarity. In this case, update rule is $z_{k+1}=z_{k}-\mu \left[\left(z_{k}+0.3\right)+\lambda z_{k}{\left({\left|z_{k}\right|}^{2}-1\right)}^{2}\right]$. In the case of (4), the algorithm works in the Riemannian space (unit circle) determined by the condition $zz^{*}=1$. At each point of $z_{k}$ the algorithm determines the search direction tangent to the unit circle and after each iteration the obtained point is projected back to the unit circle. In this case, update rule has the form $z_{k+1}=\pi \left(z_{k}-\mu \left(\frac{\partial J}{\partial z^{*}}-\langle z,\frac{\partial J}{\partial z^{*}}\rangle z\right)\right)=\pi \left(z_{k}-\mu [z_{k}\left(1-{\left|z_{k}\right|}^{2}\right)-0.3\left(z_{k}^{2}-1\right)]\right)$ where π is the projection operator on the unit circle. In Algorithm (5) we used a multiplicative optimization algorithm on the Lie group described in Section 4. In this case, the update rule has the form zk+1=exp(0.6 μiIm(zk))zk where Im(zk) is imaginary part of $z_{k}$. The starting point of each algorithm is $z_{0}=\exp\left(i\pi /4\right)$. Figure 3 shows the results of the simulation.

**Figure 4:**
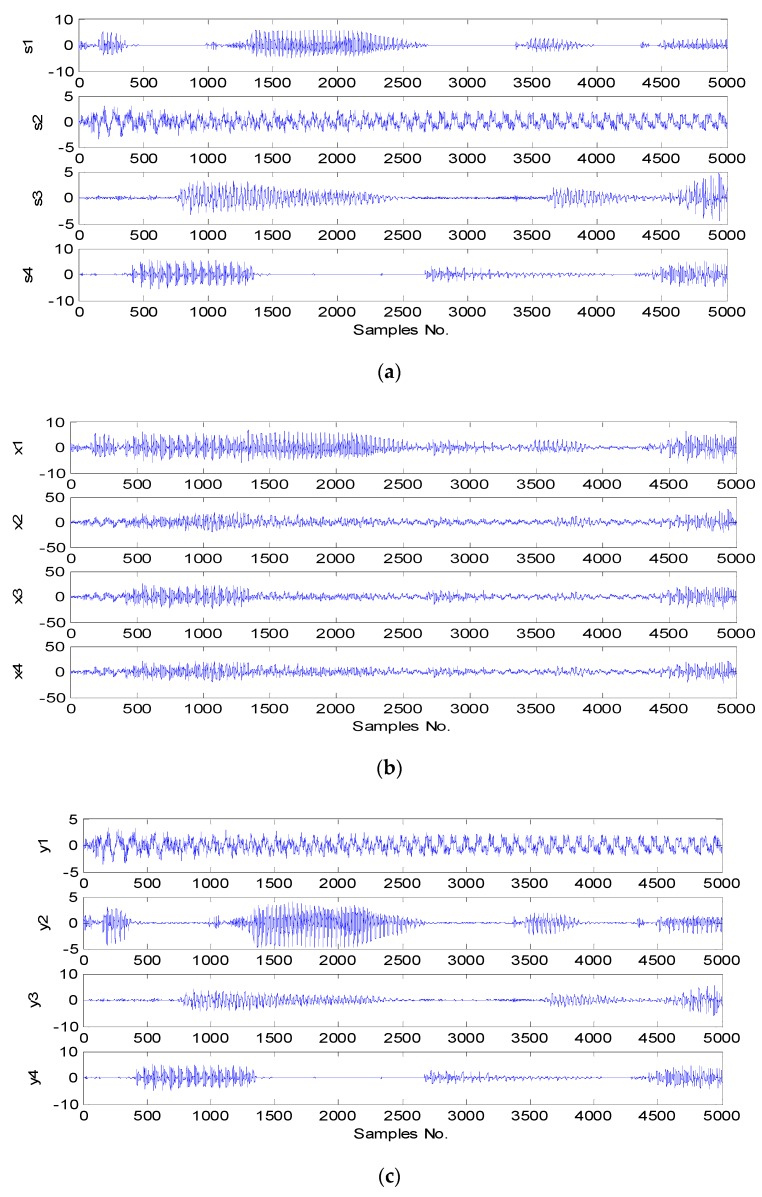

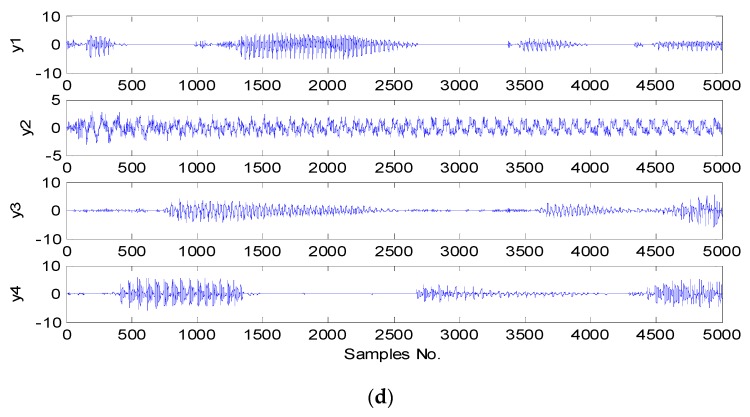
Comparison of ICA results using INFOMAX algorithm and optimization on the SO(4) group, (**a**) source signals, (**b**) observed signals (mixed), (**c**) ICA results for the INFOMAX algorithm, (**d**) ICA results for the algorithm on the group SO(4).

In the point $z_{\left(1\right)}=-0.3$ the cost function reaches its minimum $J\left(z_{\left(1\right)}\right)=0$. However, this is the undesirable minimum determined in the Euclidean space by Algorithm (1) not taking into account the unitarity constraint (Figure 3). The minimum considering this constraint is at the point $z_{min}=-1$ and the value of the cost function reaches its minimum over the Riemannian space, i.e., on the unit circle $J\left(z_{min}\right)=0.49$. Unconstrained SD Algorithm (1) and with penalty function (3) achieve undesirable minimums in points respectively from $z_{\left(1\right)}=-0.3$ and from $z_{\left(3\right)}=-0.5$ while Algorithms (2) (4) and (5) minimum appropriate $z_{min}=-1$. In the case of Algorithms (2) and (4), a characteristic “zig-zag” is associated with lowering the constraint surface and undesirable from the point of view of optimizing properties. Algorithm (4) determines the SD direction tangent to the constraint condition, thus leaving the unit circle in the optimization motion. The resulting point is again projected into a unit circle. Algorithm (5) using the multiplicative update rule on the Lie group (phase rotation) described in this article naturally ensures the condition of unitarity at each step. The optimization movement takes place at each step along the geodesic line. This simple one-dimensional example is only intended to present the idea of algorithms on Lie groups. The following is an example of using these methods on a real signal. As an example of the practical application of optimization methods on Lie groups, we will present a solution to the ICA problem. As the source signals, three speech recordings and a quasi-noisy signal (harmonic signal with high noise content) (Figure 4) with a length of 5000 samples (1.25 s) were used. The source signals were mixed using a four-by-four random mixing matrix. The four observed signals are shown in Figure 4. A SO(4) group optimization algorithm was used to implement ICA.

**Figure 5:**
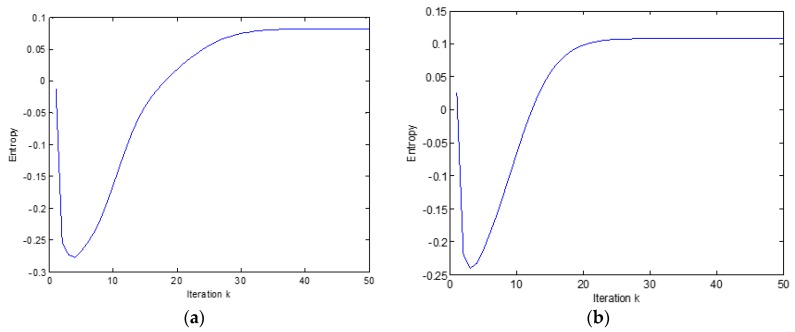
Comparison of entropy sum value of received components, (**a**) INFOMAX algorithm, (**b**) optimization algorithm on a group SO(4).

For comparison, the INFOMAX algorithm in its original form was also used [32]. Based on visual inspection and listening to the separated components, it can be concluded that ICA results using the INFOMAX algorithm and optimization on the SO(4) group are good with scale and permutation accuracy. The INFOMAX algorithm with the assumed convergence criterion converges after about 30–40 steps, while the algorithm on the SO(4) group after about 20 steps. Figure 5 shows the sum of entropy values of separated components depending on the iteration number.

The optimization algorithm on the SO(4) group converges to E(Y)=0.11 (The entropy value was determined according to an approximate relationship [32]: $E\left(Y\right)=-\underset{i}{\sum }E\left\{\underset{n}{\sum }\tan h(y_{i})\right\}+\log\left(\det\left(W\right)\right))$ while the INFOMAX algorithm to E(Y)=0.084. Listening to the results and comparison with the sources confirms the better ICA separation results obtained by the SO(4) group optimization algorithm.

## 6. Conclusions

This paper described the application of the Lie group methods for blind signal processing, including ICA and ISA. Theoretical fundamentals of the Lie groups and the Lie algebra as well as the geometry of problems occurring in BSP and basic optimization techniques based on the use of Lie groups are presented. Owing to the specific geometry and algebraic properties of BSP problems, it is possible to use Lie group methods to solve these problems. The homogeneity of search space (parameters) in BSP problems enables the use of optimization techniques based on the Lie group methods for the groups O(n) and SO(n). It has been demonstrated that the one-parameter subalgebra $so_{\Omega }\left(n\right)$ ensures the convenient property of commutating search directions. In addition, the presentation of an antisymmetric matrix (search direction) in the Jordan canonical form establishes the toral subalgebra t(p)⊂so(n), which—in terms of optimization algorithms—ensures low computational complexity and high process dynamics.

## Figures and Tables

**Figure A1:**
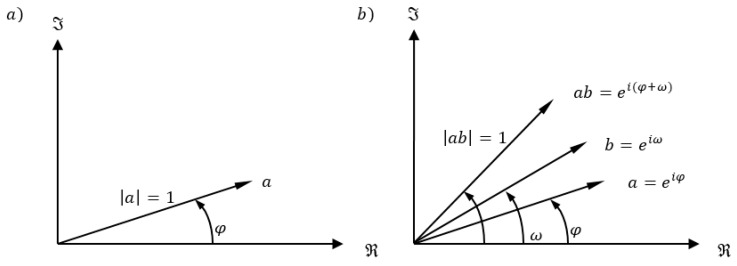
Complex value (**a**) a in r−φ notation, and (**b**) product of a and b.

## Appendix A. Lie Group and Lie Algebra

A characteristic of Lie groups is optimization motion that always leaves the orthogonality condition intact (the system constantly remains on the constraint surface). Before providing a formal definition of Lie groups, we will give an example to illustrate this concept. Let us consider a set of complex numbers of modulus 1 in r−φ notation: $G=\left\{z\in C|\left|z\right|=\left|e^{i\varphi }\right|=1,\;\varphi \in R\right\}$. If one of these numbers $a=e^{i\varphi }$ is multiplied by a number $b=e^{i\omega }$, we get $ab=e^{i\left(\varphi +\omega \right)}$, which is also several modulus 1, i.e., it belongs to a set Z (Figure A1). On the other hand, if we want to reach any number $c=e^{i\tau }$ of modulus 1 starting from the number $a=e^{i\varphi }$, it is enough to multiply a by $b=e^{i\omega }$ such that ω=τ−φ.

Thus, it can be observed that a group is formed by the set of complex numbers Z of modulus 1 combined with a multiplication operation. As a reminder, the group G is formed by a set of elements and a group operation (·) (colloquially known as multiplication) with the following properties:

1. Closure under group operation: if a,b∈G then a·b∈G
2. Associativity: a·(b·c)=(a·b)·c
3. There exists a neutral element I and an inverse element $z^{-1}\in G$ for every element of the group, such that: $I\cdot z=z\cdot I=z.\;z\cdot z^{-1}=z^{-1}\cdot z=I$

It can easily be verified that the set Z satisfies these conditions with a group operation in the form of multiplication of complex numbers, the neutral element $I=1=e^{i0}$ and the inverse $z^{-1}=e^{-i\varphi }$ of any element of $z=e^{i\varphi }$. Moreover, since the multiplication operation in the set G is commutative, this set forms a commutative group, also known as an abelian group. In addition, the group G is smooth, i.e., differentiable. We can perform an infinitesimally small movement in the group by going from one number to another, these numbers differing from each other by an infinitesimally low value. In fact, the group G locally “looks” like a section of the real line R. This means that the motion in the group G can be described with one parameter, e.g., the angle φ, and thus this group with the coordinate system of φ creates a differential manifold. The group which is a smooth differential manifold is called the Lie group. The Lie group combines algebraic properties of the groups possessing the properties of a smooth differential manifold. As a result, we can, for example, consider a tangent space $T_{x}G$ of the Lie group G at point x. In a special case when x=I, the tangent space in an identity element of the group $T_{I}G$ additionally provided with an elementary operation known as the Lie bracket is called the Lie algebra g. The group operation (multiplication) by a constant element of the group Q creates a differentiable mapping of the group G to itself, i.e., a diffeomorphism known as left translation (A1):

(A1) $$L_{Q}:\;G\to G:\;W\to QW;Q,W\in G$$

and right translation (A2):

(A2) $$R_{Q}:\;G\to G:\;W\to WQ;Q,W\in G$$

and tangential mappings induced by them (A3) and (A4):

(A3) $$dL_{Q}:\;T_{W}G\to T_{QW}G:\;Z\to QZ;Z\in T_{W}G,\;Q\in G$$

(A4) $$dR_{Q}:\;T_{W}G\to T_{WQ}G:\;Z\to ZQ;Z\in T_{W}G,\;Q\in G$$

A characteristic of the Lie group is that each of its elements W can be “moved” by left or right translation to a “convenient” neighborhood of the identity element, and the same applies to every tangent space $T_{W}G$:

(A5) $$L_{W^{-1}}\left(W\right)=W^{-1}W=I\;:R_{W^{-1}}\left(W\right)=WW^{-1}=I$$

(A6) $$dL_{W^{-1}}\left(T_{W}G\right)=\left\{W^{-1}Z=X\in T_{I}G=g\right\}\;:dR_{W^{-1}}\left(T_{W}G\right)=\left\{ZW^{-1}=X\in T_{I}G=g\right\}$$

Equation (A6) can also be written in a reversed form (A7):

(A7) $$T_{W}G=dL_{W}\left(T_{I}G\right)=\left\{WX;\;X\in T_{I}G=g\right\}:\;T_{W}G=dR_{W}\left(T_{I}G\right)=\left\{WX;\;X\in T_{I}G=g\right\}$$

From the above it can be seen that the tangent space $T_{W}G$ via the tangent mapping $dL_{W^{-1}}$ or $dR_{W^{-1}}$ is transferred to the Lie algebra g.

From the above one can draw an important conclusion. If the structure of a Lie algebra g is known, it can be used to conveniently parameterize the neighbourhood of an identity element of the group G via the application of a suitable homeomorphism. Such homeomorphism is widely known as the exponential map or, in short, exponentiation, and is denoted as exp : g→G. It should be noted that “exp” is here only a symbolic denotation and even in the case of Lie matrix groups it does not necessarily mean a matrix exponentiation operation.

A set of 2-dimensional orthogonal matrices $W^{T}W=I_{2}$ forms a Lie group as is done by the set of complex numbers of modulus 1. It is also an abelian (commutative) group and has the same algebraic properties as the set of complex numbers of modulus 1. In general, square *n*-dimensional orthogonal matrices forming the group O(n) can be presented in a block-diagonal form with diagonal elements as 2×2 orthogonal matrices. This form is known as the Jordan canonical form of orthogonal matrix [23]. Understanding the “behaviour” of the Lie groups over the complex numbers of modulus 1 and thus 2×2 orthogonal matrices is therefore crucial for analysis of a general case. The group O(n) is also a Lie group composed of two disjoint parts (subsets): the orthogonal matrices with determinant 1 and the matrices with determinant −1. For example, for the group O(2), these two components can be shown in the form (A8):

(A8) $$W=\left(\begin{array}{cc} cos\varphi & sin\varphi \\ -sin\varphi & cos\varphi \end{array}\right)\;W^{\prime }=\left(\begin{array}{cc} cos\varphi & -sin\varphi \\ -sin\varphi & -cos\varphi \end{array}\right)$$

$$detW=1\;detW^{\prime }=-1$$

for any real number φ that can be a Cartesian coordinate system identified with the angle of rotation. As one cannotice, both in the first and the second part, the transition from one element to another takes place (smoothly) by changing the parameter φ. The transition between the matrices of both parts is only possible via multiplying the matrices belonging to both parts, which is tantamount to permutation of the matrices. The transposition of the matrix columns or rows in any part will lead to a change in the sign of the determinant. This occurs as a transition between the parts. However, a smooth transition between the parts is not possible. Hence, these components (parts) are called disjoint. The subset of matrices with determinant 1 is called a special orthogonal group SO(n). It is a subgroup O(n) and constitutes an associative Lie group (it is possible to move between elements of the subgroup smoothly by performing multiplication in the subgroup).

In ICA problems, due to permutation ambiguity (Section 2), optima of the cost function must be present in both subgroups O(n). We will therefore limit our considerations to the (associative) subgroup SO(n) considerations, which will not diminish the general nature of the considerations. The multiplication of the matrices belonging to SO(2):

(A9) $$WV=\left(\begin{array}{cc} cos\varphi & sin\varphi \\ -sin\varphi & cos\varphi \end{array}\right)\left(\begin{array}{cc} cos\theta & sin\theta \\ -sin\theta & cos\theta \end{array}\right)=\left(\begin{array}{cc} \cos\left(\varphi +\theta \right) & sin\left(\varphi +\theta \right)\\ -sin\left(\varphi +\theta \right) & cos\left(\varphi +\theta \right) \end{array}\right)$$

This involves, as one can observe, adding the angles φ and θ. The above also demonstrates that the multiplication operation in the group SO(2) is commutative. The group SO(2) is also known as the rotation group because its operation over any vector can be associated with a rotation on the plane $R^{2}$. Moreover, the group SO(2)—like the group of complex numbers of modulus 1 can be parameterized by means of one parameter—the angle 0≤φ<2π. Groups that “operate” in the same way (have the same algebraic properties) are called isomorphic. From the point of view of optimization techniques, the key question is how to determine a derivative in the Lie group G. Basing on the previously considered Lie group of complex numbers of modulus 1, let us consider the curve $z\left(t\right)=e^{i\varphi \left(t\right)}$ in the group G parameterized by t (the t parameter can be identified as time). Assuming that φ(t)=ωt, the derivative of the curve z(t) with respect to t has the form (A10):

(A10) $$\frac{dz}{dt}=\frac{d}{dt}\left(e^{i\varphi \left(t\right)}\right)=i\left(\frac{d\varphi }{dt}\right)e^{i\varphi \left(t\right)}=i\omega e^{i\varphi \left(t\right)}=i\omega z$$

As one can observe, the derivative z(t) is proportional to $iz=e^{i\frac{\pi }{2}}z=e^{i\left(\varphi \left(t\right)+\frac{\pi }{2}\right)}$, which means that it is perpendicular to z. This result is analogous to the relationship between the vector of velocity and the radiusin circular motion. Assuming that ω is an angular velocity and z is a radius, the circular motion can be described in an identical manner. The velocity vector of length ωz is tangent to the trajectory, i.e., it is perpendicular to the radius of the circle. A similar relationship can be obtained by differentiating the group SO(2). Likewise, assuming that φ=ωt and differentiating $W=\left(\begin{array}{cc} cos\varphi & sin\varphi \\ -sin\varphi & cos\varphi \end{array}\right)$ relative to t, we get (A11):

(A11) $$\frac{dW}{dt}=\frac{d}{dt}\left(\begin{array}{cc} cos\varphi & sin\varphi \\ -sin\varphi & cos\varphi \end{array}\right)=\left(\begin{array}{cc} -sin\varphi & cos\varphi \\ -cos\varphi & -sin\varphi \end{array}\right)\omega =\left(\begin{array}{cc} 0 & \omega \\ -\omega & 0 \end{array}\right)W=XW$$

One can notice that similarly to the group of complex numbers of modulus 1, the derivative in the group SO(2) is proportional to an element of the group where the derivative dW/dt~W is determined. It is easy to check that this derivative is tangent at point W to the constraint surface $tr\left({\left(\frac{dW}{dt}\right)}^{T}W\right)=trX=0$. This pattern is also true for the general case SO(n). Differential equations with the structure $\frac{dA}{dt}=B\left(t\right)A\left(t\right)$, where A(t)∈G belongs to the Lie group and B(t)∈g belongs to the Lie algebra, are known as differential equations of the Lie groups. As with the solution of the scalar differential equation $\frac{dz}{dt}=az⟹z\left(t\right)=e^{at}$, the solution of the differential Equation (A11) is a matrix W(t)=exp(tX), where exp(.) is a matrix exponentiation operation given by the general formula (Maclaurin series) (A12):

(A12) $$\exp\left(tX\right)=I+tX+\frac{t^{2}X^{2}}{2!}+\ldots +\frac{t^{k}X^{k}}{k!}+\ldots ,$$

It can easily be verified that W(t) satisfies Equation (A11):

$$\frac{dW}{dt}=\frac{d}{dt}\exp\left(tX\right)=X\exp\left(tX\right)=XW$$

The space tangent to SO(2) at point W is given by the matrices, where $X=-X^{T}=\left(\begin{array}{cc} 0 & \omega \\ -\omega & 0 \end{array}\right)$ is an antisymmetric matrix. As one can observe, with any antisymmetric matrix X≠0, every element of the group SO(2) can be made precise by defining a single parameter t as (A13):

(A13) $$W\left(t\right)=\exp\left(tX\right)=\exp\left(\begin{array}{cc} 0 & t\omega \\ -t\omega & 0 \end{array}\right)=\exp\left(\Omega \right)$$

where $\Omega =-\Omega^{T}=\left(\begin{array}{cc} 0 & t\omega \\ -t\omega & 0 \end{array}\right)$ is an antisymmetric matrix.

From the point of view of the optimization process, it is a very convenient property. Instead of searching in the space of orthogonal matrices, it is enough to search in the space of the angles φ or, alternatively, in the space of antisymmetric matrices Ω. The space tangent to SO(n) in the identity (neutral) element $I_{n}$ is therefore determined by the set of antisymmetric matrices $\Omega =-\Omega^{T}$. According to previous general considerations, this space is called a Lie algebra so(n) of the Lie group SO(n). Homeomorphism is determined by the matrix exponential operation, Ω∈so(n)→exp(Ω)∈SO(n) (or exp:so(n)→SO(n)). As mentioned above, from the point of view of the optimization process, it is convenient to navigate in the Lie algebra because it is a vector space, so the addition of elements and multiplication by scalars are allowed. The result of such operations will still belong to the Lie algebra. The idea presented above for the group SO(2) is generalized to SO(n). However, for n≥3, the matrix multiplication is not commutative: exp(A)exp(B)≠exp(B)exp(A). From the general dependence (A14):

(A14) $$\exp\left(\varepsilon A\right)\exp\left(\varepsilon B\right)-\exp\left(\varepsilon B\right)\exp\left(\varepsilon A\right)=\varepsilon^{2}\left[A,B\right]+O\left(\varepsilon^{3}\right)$$

where [·,·] is the matrix commutator or the Lie bracket defined as [A,B]=AB−BA, $O\left(\varepsilon^{3}\right)$ is a lower order $\varepsilon^{3}$ and ε≪1 is a scalar, it follows that the “non-commutativeness” of matrix multiplication in the group SO(n) is expressed with the commutator [·,·] of the matrices belonging to the Lie algebra so(n). The commutator of antisymmetric matrices is also an antisymmetric matrix ${\left[A,B\right]}^{T}={\left(AB-BA\right)}^{T}=B^{T}A^{T}-A^{T}B^{T}=BA-AB=-\left[A,B\right]$, which means that [A,B]∈so(n). The Lie algebra of antisymmetric matrices is therefore closed under addition, multiplication by scalars and the Lie bracket. In addition to this, the Lie bracket has the following properties (A15)–(A17):

(A15) [A,A]=0

(A16) [A+B,C]=[A,C]+[B,C]

(A17) [A,[B,C]]+[B,[C,A]]+[C,[A,B]]=0

The first two properties result from the antisymmetric nature of the commutator, while the third property is called the Jacobi identity and is widely known in differential geometry. The multiplication of 2×2 antisymmetric matrices A,B∈so(2) is commutative, therefore [A,B]=0, and hence SO(2) is an abelian group. Elements of the group SO(3) can be equated with the rotations relative to the coordinate system origin of the space $R^{3}$. As we know, such rotations are not commutative. Therefore, the group SO(3) is non-abelian (non-commutative).
